# Integration of Bulk RNA-seq Pipeline Metrics for Assessing Low-Quality Samples

**DOI:** 10.21203/rs.3.rs-6976695/v1

**Published:** 2025-07-03

**Authors:** Samuel Hamilton, Gaurav Gadhvi, Tyler Therron, SCRIPT Investigators, Deborah R. Winter

**Affiliations:** 1 Department of Medicine, Division of Rheumatology, Feinberg School of Medicine, Northwestern University, Chicago, IL, USA; 2 Center for Human Immunobiology, Feinberg School of Medicine, Northwestern University, Chicago, IL, USA

**Keywords:** Quality Control, RNA-seq, open-source software, machine learning, technical bias

## Abstract

**Background:**

With the rise of RNA-seq as an essential and ubiquitous tool for biomedical research, the need for guidelines on quality control (QC) is pressing. Specifically, there remains limited data as to which technical metrics are most informative in identifying low-quality samples.

**Results:**

Here, we addressed this issue by developing the Quality Control Diagnostic Renderer (QC-DR), software designed to simultaneously visualize a comprehensive panel of QC metrics generated by an RNA-seq pipeline and flag samples with aberrant values when compared to a reference dataset. As an example, we applied QC-DR to the Successful Clinical Response in Pneumonia Therapy (SCRIPT) dataset, a large clinical RNA-seq dataset of sequenced alveolar macrophages (n = 252). Next, we used this dataset to assess relationships between a variety of QC metrics and sample quality. Among the most highly correlated pipeline QC metrics were *%* and *# Uniquely Aligned Reads*, *% rRNA reads*, *# Detected Genes*, and our newly developed metric of *Area Under the Gene Body Coverage Curve (AUC-GBC*), while experimental QC metrics derived from the lab were not significantly correlated. We then trained a set of machine learning models on the SCRIPT dataset to evaluate the relative contribution of QC metrics to sample quality prediction. Our model performs well when tested on an independent dataset despite differences in the distribution of QC metrics.

**Conclusions:**

Our results support the conclusion that any individual QC metric is limited in its predictive value and suggests approaches based on the integration of multiple metrics with QC thresholds. In summary, our work provides new insights, practical guidance, and novel QC software which can be used to improve the methodological rigor of RNA-seq studies.

## Introduction:

Over the past couple decades, RNA-seq has emerged as a key technology for translational research in the biomedical field. However, while quality control (QC) is fundamental to RNA-seq analysis, performing rigorous QC remains challenging. (1–3). Although RNA-seq does not suffer from many of the quality problems that hindered preceding microarray technologies – such as poor throughput, signal saturation, and background signal – low-quality samples are nevertheless common (4–7). Many variables affecting quality, including cell number, viability, and time to processing, are difficulty to optimize when using patient samples whose availability is subject to clinical priorities (8). Low-quality RNA-seq samples are especially disruptive because: 1) limited biological material prevents the collection of technical replicates, 2) high variability between patients complicates the differentiation of technical noise from true biological signal, and 3) experimental designs that minimize technical effects are often not feasible (9–12). Rapid reduction of sequencing costs and rise in publicly available data have increased the scale of RNA-seq experiments, which has made screening samples individually for quality more laborious (13). This issue is especially pertinent to core facilities, large consortium projects, and laboratories performing meta-analyses on publicly available data from repositories such as UK Biobank, The Cancer Genome Atlas, and the ENCODE project (14–16). In short, the developments that have made RNA-seq data a fundamental and ubiquitous tool in biomedical research have also made their QC more challenging.

Typically, researchers will perform “wet lab” tests to assess prior to sequencing to determine which samples should proceed to sequencing. These include assessing cell number and viability prior to RNA extraction as well as determining RNA/cDNA concentration at later steps in library preparation (17). It has also become common to calculate the RNA integrity number (RIN) by quantifying the ratio of 28S and 18S rRNA using automated gel electrophoresis instruments such as the 210 Agilent Bioanalyzer or the Agilent 4200 Tape Station RNA (18–21). However, RIN cannot be calculated on low concentration input, and, when using patient samples that cannot be replicated, researchers may choose to risk sequencing samples regardless of experimental QC values rather than abandon precious data points. Moreover, performing QC on these publicly available data can be challenging as important experimental information, such as the initial sample yield and data on RNA quality are not typically provided. Thus, it is critical to be able to interpret sample quality from post-sequencing QC derived from RNA-seq pipelines.

After sequencing, raw RNA-seq data must be run through computational pipelines to convert it into a format ready for analysis. Pooled libraries typically generate data stored as a BCL file, which is first demultiplexed into individual FASTQ files for each sample. These FASTQ files contain sequencing quality information for each base of every read which can be summarized using tools such as FASTQC and HTQC (22). These tools also report on statistics including GC content, base quality score, transcript length, adapter content, and the number of overrepresented sequences (23,24). Next, most pipelines include a trimming step where software such as Trimmomatic, BBDuk, or Trimgalore remove sequencing adapters and low-quality reads from the FASTQ files (25–28). Trimmed FASTQ files are used as inputs for aligners, such as HISAT2, STAR, or TopHat (29,30), which produce SAM files (sequence alignment map or BAM if in binary) which provide information on where each read maps with in the genome. Finally, reads are quantified per gene typically by overlapping exons of the transcript and counted. Common tools for the gene quantification step include HTSeq2 and featureCounts (31,32). Many metrics can be derived directly from the various steps of the pipeline or easily calculated with simple functions. However, the key challenge is understanding how to best synthesize these numerous pipeline metrics together into a comprehensive QC analysis.

Currently, there is no community standard for defining low-quality samples in an RNA-seq dataset. The percent of aligned reads is commonly used for sample quality but there is no objective way to set a threshold. Moreover, a sample with low aligned reads may still be usable if it has a high absolute number of aligned reads and a sample with high aligned reads may still suffer from ribosomal (rRNA contamination) and/or low library complexity. Principal component analysis (PCA) is frequently used to visualize the dataset in reduced dimensional space and identify outliers by eye. As datasets grow larger, noise, batch, and biological variability reduce the effectiveness of this approach, and very obvious outliers may obscure the presence of more subtle but still problematic samples. Popular QC visualization software, including FastQC, FaQC, PRINSEQ, can assist users by producing plots for each sample summarizing metrics, such as average base quality and reads length, and flagging concerning samples (23,33,34). However, they solely focus on metrics obtained from FASTQ files rather than those generated by the full pipeline and the flags are based on specific assumptions about the technology being used: they are typically based on whole genome sequencing rather than transcriptome-specific. Alternatively, software such as RaNA-Seq, MultiQC, and QuaCRS produce visualizations that show a wide distribution of QC metrics across processing steps but produce a separate report for each metric without identifying outliers, which makes it difficult to identify poor-quality samples that present with consistently poor QC values (35–37). The RSeQC package includes a number of useful tools including an algorithm to calculate and visualize average base coverage across genes in a RNA-seq sample (38,39). This output is meant to reflect RNA integrity and is particularly useful for full-length protocols; it may be less effective at detecting low-quality samples from end biased protocols or those with contamination and/or low depth. In summary, an RNA-seq QC visualization tool that both provides a range of metric across different steps of the pipeline and allows comparison within the dataset would better inform researchers to identify low-quality samples.

To address these issues, we present the Quality Control Diagnostic Renderer (QC-DR), which produces Integrated visualizations of many pipeline QC metrics simultaneously, showing each sample within the context of a reference dataset. We demonstrate the use of QC-DR on a large clinical dataset where it identifies samples that require further examination. Next, we perform a systematic analysis of the relationships between QC metrics and sample quality. Finally, we build a machine learning model to investigate how different QC metrics can be used in combination to reflect sample quality and test it on an independent dataset. Our results not only provide the community with a novel software to identify low-quality samples but also guidance on which pipeline metrics are most informative in setting QC thresholds for filtering samples.

## Results:

### QC-DR Facilitates a Comparison of Multiple RNA-seq Pipeline QC Metrics Across Samples.

**Figure 1.**
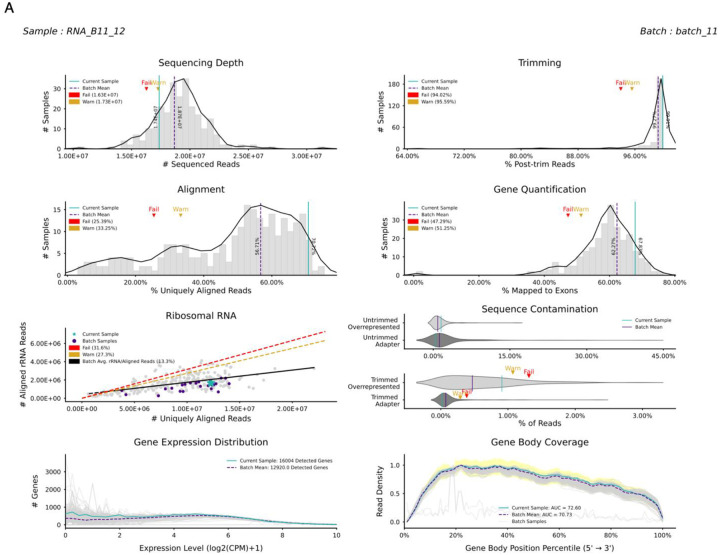
QC-DR Overview. **(A)** An example of a QC-DR sample report: *Sequencing Depth, Trimming, Alignment, Gene Quantification*: Histograms show the distribution across all samples in the reference dataset. The black line shows the smoothed kernel density of the distribution. *Ribosomal RNA:* The scatterplot shows the *# Uniquely Aligned Reads* and *# rRNA Reads* for samples in the query and reference datasets. The black line shows the least squares regression line. *Sequence Contamination:* The violin plots show the smoothed distribution of overrepresented and adapter sequences pre- (top) and post- (bottom) trimming. *Gene Expression Distribution:* Each curve represents the histogram of the gene frequency at given expression levels (binned by 0.25). *Gene Body Coverage:* Each curve represents the read density is calculated by percent of length for each gene, scaled, and then averaged across all genes. Highlighted area indicates denotes the 95% confidence interval at each percentile. For all plots, the dashed purple line represents the mean of the current batch (except in *Ribosomal RNA*, where each point is a batch sample). The teal line (star in *Ribosomal RNA)* indicates the current sample. The thresholds for fail (red) and warn (yellow) are indicated.

We developed QC-DR as a comparative RNA-seq QC analysis tool which visualizes how a sample performs across multiple QC metrics derived from an RNA-seq pipeline compared with other samples in the same dataset or a given reference (Fig. S1A–B, Extended Data 1). QC-DR has both a Unix and Python interface, is open-source, and is available on GitHub (40). From a query table of QC metrics as input (Supplementary Table 1), QC-DR produces a report for each sample with up to eight subplots illustrating how QC metrics compared to samples in the rest of the query or in the optionally supplied reference dataset (Fig. 1A, S1C). The first four subplots produced by QC-DR assess metrics across the different stages of RNA-seq processing pipeline: *# Sequenced Reads* reflecting Sequencing Depth, *% Post-trim Reads* reflecting Trimming, *% Uniquely Aligned Reads* reflecting Alignment, and *% Mapped to Exons* reflecting Quantification (Fig. 1B, Table 1). The fifth and sixth subplots generated by QC-DR quantify the ribosomal RNA (rRNA) fraction and sequence contamination (through adapters and overrepresented sequences), respectively. The seventh plot creates a multi-sample histogram based on an optional table of gene expression distributions for each sample (Supplementary Table 2). The eighth subplot shows the average 3’ -> 5’ coverage across all genes based on another optional table (Supplementary Table 3). These 2 optional tables can be generated by the user or through QC-DR given the gene expression table (for the histogram) and a folder of BAM files (for the gene coverage). The seventh and eighth plots provide intuitive visualizations of library complexity and RNA integrity that are widely used in the field. To quantify these measures, we propose two novel metrics: *# Detected Genes* and *Area Under the Curve of Gene Body Coverage (AUC GBC)*.

In each subplot, the value of the current sample is highlighted in teal, while the “batch” mean (or all samples in the batch for the rRNA plot) is indicated in purple. Users can specify subsets of samples within the dataset as batches; otherwise, the whole query is treated as one batch. Other samples in the user dataset, or in the reference if one is given, form the background and are shown in grey. For each subplot, QC-DR identifies samples that deviate from the reference, assigning “Warn” or “Fail” flags, which are displayed in each sample’s report. As the default, Warn and Fail cutoffs are set to statistical thresholds of 0.1 and 0.05, respectively, but the users can adjust these thresholds or define custom cutoff values to tailor the flags to their specific research needs; in either case, the absolute cutoffs are provided along with the output (Supplementary Table 4). The aggregated “Warn” and “Fail” flags are presented in a summary heatmap (Fig. S1B) and an Excel table (Supplementary Table 5), enabling rapid identification and further examination of low-quality samples. Taken together, these subplots provide a holistic assessment of each sample. In this way, QC-DR streamlines and enhances the comprehensive QC process for large RNA-seq datasets.

### QC-DR Flags Are Associated with Technical Bias in a Large Clinical RNA-seq Dataset.

**Figure 2.**
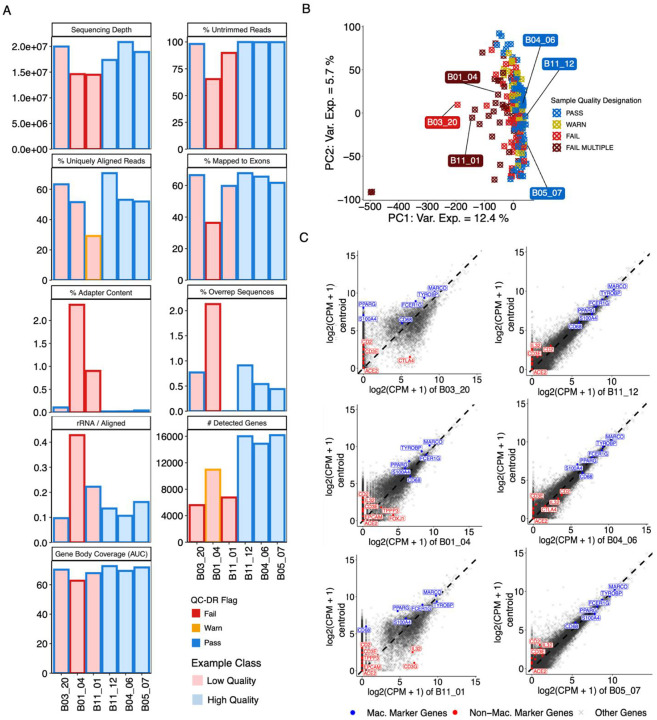
Examples of Low-quality Samples in SCRIPT Dataset. **(A)** Bar plots displaying QC-DR metrics for three example low-quality samples (pink) from the SCRIPT dataset, which received at least one fail flag, as well as three example high-quality samples (light blue), which received no flags. Bar outlines indicate whether the metric was a pass (blue), warn (yellow), or fail (red). **(B)** PCA of gene expression values for SCRIPT dataset where samples are colored depending on whether they did not receive a flag for any metric (blue), only received warn flags (yellow), received a single fail flag (red), or received multiple fail flags (dark red). **(C)** Scatterplots showing gene expression values from low-quality and high-quality example samples compared against a centroid generated by averaging all other samples. Macrophage marker and non-macrophage marker genes are labeled and colored red and blue, respectively.

To demonstrate how QC-DR can be used to identify low-quality samples in a large dataset, we input QC data from 252 alveolar macrophage RNA-seq samples collected from mechanically intubated patients enrolled in the Successful Clinical Response in Pneumonia Therapy (SCRIPT) project (41). We used the default mode for cutoffs which flags samples in the extreme 10% (warn) and 5% (fail) of sample distributions. We identified 102 samples (40%) that “failed” a minimum of one QC metric in QC-DR (Fig. S2A). We chose three examples that failed at least one metric to explore further in comparison with three “high quality” samples that passed all metrics (Fig 2A). Sample B03_20 passed all categories with the exception of *# Detected Genes*. On the other hand, Sample B01_04 was flagged in every category except *% Uniquely Aligned Reads*. Finally, Sample B11_01 had a mixture of passes, warns, and fails; notable, this samples passes on *% Mapped Exons* and *Gene Body Coverage*, while failing on *# Detected Genes*. Next, we visualized all samples in the dataset based on principal component analysis (PCA) of their gene expression values (Fig. 2B). As is commonly seen in RNA-seq analyses, all of the outliers are samples flagged by QC-DR, suggesting that they are the result of technical bias. However, we also note that not all samples flagged by QC-DR are outliers on the PCA. For this dataset, we had access to multiple experimental QC measured during the course of sample processing (Fig S2B, Table 1). Interestingly, while our 3 examples are among outliers in the PCA, they generally do not stand out based on experimental QC, though B1_04 does have low RNA fragment size (Fig S2C). To further characterize how our chosen examples differ from the average sample in the dataset, we calculated the dataset centroid for each gene. When plotted against the centroid, the 3 failed examples demonstrated lower correlation, particularly for lowly expressed genes, with reduced expression of canonical macrophage markers and increased expression of genes associated with other cell types (Fig 2C). While QC-DR effectively highlights potentially low-quality samples with no *a priori* cutoffs, a more sophisticated schema is required to further distinguish the samples that are truly low quality from the false positives.

### No Individual QC metric is a Reliable Indicator of Low Quality.

**Figure 3.**
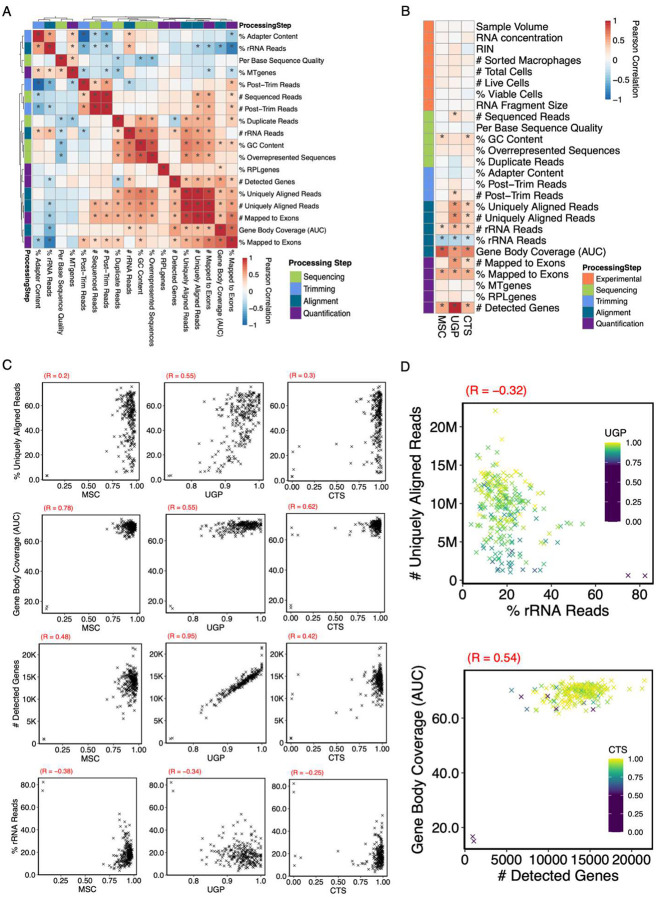
Relationship of Experimental QC, Pipeline QC, and Endpoint Metrics. (A) Heatmap showing the Pearson correlations between pipeline QC metrics in the SCRIPT dataset. (B) Heatmap showing the Pearson correlation between experimental and pipeline QC versus endpoint metrics in the SCRIPT dataset. (C) Scatterplots depicting select pipeline QC versus endpoint metrics in the SCRIPT dataset. The correlation coefficient is given on the top left of each plot. (E) Scatterplots showing the relationships between two pipeline QC metrics with an added color scale that shows an endpoint metric in the SCRIPT dataset. The correlation coefficient is given on the top left of each plot. For all plots, * indicates statistically significant correlations after multiple hypothesis testing correction (p adj. < .05)

To better understand how various QC metrics reflect sample quality, we further examined a more extensive list of 18 Pipeline QC metrics from the SCRIPT dataset that can be derived from the RNA-seq processing pipeline directly or with small adjustments (Table 2, Supplementary Table 7). Not surprisingly, we found several clusters of closely correlated metrics, such as the group including *% Uniquely Aligned Reads, # Uniquely Aligned Reads, and # Mapped Exons* (Fig. 3A). Similarly, some, but not all of the QC metrics, were associated with Experimental QC values: the previously mentioned group was moderately correlated with RNA concentration and sorted macrophages (Fig. S3A, Supplementary Table). Interestingly, *RNA Fragment Size* had the most variable relationship across QC metrics with significant positive correlation to *% Mapped to Exons*, almost zero correlation to *% Uniquely Aligned Reads*, and significant negative correlation to *%rRNA Reads* (Fig. S3B).

Next, we compared the QC metrics to endpoint metrics designed to assess the quality of these samples based on the known characteristics of this dataset. These include *(1)* Mean Sample Correlation (MSC), which assesses how aberrant a sample’s RNA-seq profile is compared to the average sample; (2) Cell Type Score (CTS), which indicates how strongly a sample expresses canonical macrophage marker genes compared to other cell type markers (Supplementary Table 7); and (3) Unique Genetic Positions (UGP), which measures the complexity of the library based on the number of based covered in the genome (Fig. S3C, Supplementary Table 6). These endpoint metrics exhibit only a moderate degree of correlation with each other indicating that they each reflected distinct aspects of RNA-seq sample quality (Fig. S3D–E). We found that many of the metrics were significantly correlated with one or more endpoint metrics (Fig. 3B–C). The previously mentioned group including *% Uniquely Aligned Reads* were moderately correlated with both UGP and CTS; though the relationship appears to be non-linear. The highest positive correlations were observed between *AUC-GBC* vs. MSC and # *Detected Genes* vs. UGP. On the other hand, *% rRNA Reads* was the only metric that was significantly negatively correlated with endpoint metrics. Notably, no Experimental QC values were significantly correlated with any of the endpoint metrics (Fig. 3B). While samples with the lowest endpoint metric values tended to have a RIN that was not calculable, many of these samples were high quality (Fig. S3F). Similarly, *RNA concentration* and *RNA Fragment Size* were poor predictors of samples quality as assessed by the endpoint metrics (Fig. S3G). QC metrics associated with endpoint metrics did not necessarily correlate with each other: *% rRNA Reads* and *% Uniquely Aligned Reads* were negatively correlated with samples high in the former and/or low in the latter scoring poorly in endpoint metrics (Fig. 3D). Moreover, samples that were low in either # *Detected Genes* or *AUC-GBC* were equally likely to be low quality as those that were low in both (Fig. 3D). Thus, no one QC metric fully captures the range of low-quality samples in a dataset.

### Integrating Multiple Metrics Leads to a Better Classification of Sample Quality.

**Figure 4.**
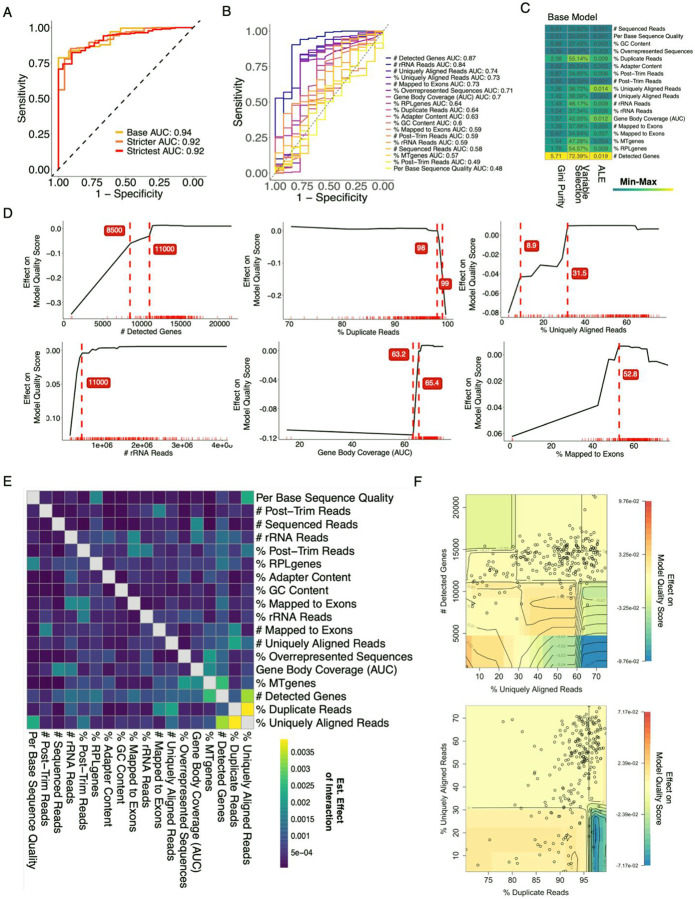
Classifying RNA-seq Quality with a Random Forest Model using Pipeline QC Metrics. (**A)** Out-of-bag (OOB) Receiver Operating Curves (ROCs) for the base, stricter, and strictest random forest models trained on the SCRIPT dataset **(B)** ROCs of single variable logistic regression models, evaluated using 5-fold cross validation on the SCRIPT dataset. (**C)** Heatmap showing variable importance scores for each pipeline QC metric in the base model based on Gini Purity, Variable Selection, and Accumulated Local Effects (ALE). Cells are colored based on the min-max normalized estimated effect, while the absolute values are given within the cell. (**D)** ALE plots for select QC metrics in the base model. Red ticks represent the actual sample values in the training set, while the black line indicates the estimated effect on the model quality score at the given value. Red dashed vertical lines indicate key inflection points within the ALE estimated model, with the values of those points labeled in red. Negative effects decrease the quality score, while positive effects increase the quality score (1=high, 0=low). **(E)** Heatmap showing the estimated effect of variable interactions on the model quality score in the base model. **(F)** Two-dimensional ALE plots showing the estimated effect of the interactions between select variables on the base model quality score. Circles represent the actual sample values.

The absence of a single variable reliably associated with sample quality and the prevalence of non-linear relationships between QC metrics and quality suggested a model incorporating multiple QC metrics would be more successful. We therefore decided to train a random forest model to predict sample quality based on the QC metrics in the SCRIPT dataset. We chose to train a random forest because of their ability to model non-linear relationships, minimal required preprocessing, and ease of interpretation (42,43). To reflect a range of QC stringency, we set three different cutoffs for each of the endpoint metrics and defined low-quality samples at each level based on those that were below the cutoff in at least two endpoints (Fig. S4A). Thus, out of the 252 SCRIPT samples, we classified 13, 18, and 44 samples (5%, 7%, and 17% of the total) as low quality at the base, stricter, and strictest levels, respectively. (Fig. S4B). We then trained three different random forest models based on these training sets (Supplementary Table 8). When predicting out-of-bag (OOB) samples (i.e. samples not used in training individual trees), the area under the receiver operating curve (AUC) was 0.92 or above for all three models (Fig 4A). This is a meaningful improvement from models based on a single QC metric where the *# Detected Genes* was the most accurate with an AUC of 0.87 (Fig. 4B). In fact, *# Detected Genes* was the single most important variable in all 3 models by all metrics we calculated (Fig. 4C, S4C). This finding aligns with our earlier observation that the *# Detected Genes* was highly and significantly correlated with all endpoint metrics. Other QC metrics – including *% Duplicate Reads, % Uniquely Aligned Reads, # rRNA reads, and AUC-GBC*, varied in variable ranking importance between models. However, the different models still performed well when predicting the other classifications (Fig. S4D). Many of the QC metrics contributing to the model are estimated to have a binary effect on the prediction where values above or below a certain value appear to be detrimental to sample quality (Fig. 4D). For example, marginal improvements in quality diminished after approximately 10,000 *# Detected Genes* and *AUC-GBC* had a precipitous climb in quality at just over 60.

To characterize how different QC metrics might interact in modelling sample quality, we adopted a two-dimensional Accumulated Local Effects (ALEs) approach, which estimates the effect on quality score for a pair of two variables within a given machine learning model. When applied to our sample quality models, this approach identified several high effect interactions that were not obvious previously and differed slightly between models (Fig. 4F, S4E). Although *% Uniquely Aligned Reads* was not estimated to have a particularly high effect on its own, it apparently interacted with *# Detected Genes* in the base model. Specifically, samples with high values in one metric but low in the other were more likely to be flagged as “low quality” that those that were low in both (Fig. 4G). This seemingly paradoxical relationship may reflect the identification of samples with either low complexity or high noise. Similarly, samples with low *% Uniquely Aligned Reads* and high *% Duplicate Reads* were also flagged (Fig. 4H). In the case of Gene Body Coverage, a slightly low *AUC-GBC* can be overlooked as long as the *# Sequenced Reads* is not above a certain threshold (Fig. S4F). Not surprisingly, the estimated quality score from the base model highlights samples that would not be identified as outliers from a visual inspection of the PCA (Fig. S4G). Overall, the performance of our random forest models suggests that multiple QC metrics and their interactions should be taken into consideration when classifying low-quality samples.

### Validation of the Sample Quality Classification on an Independent Test Set.

**Figure 5:**
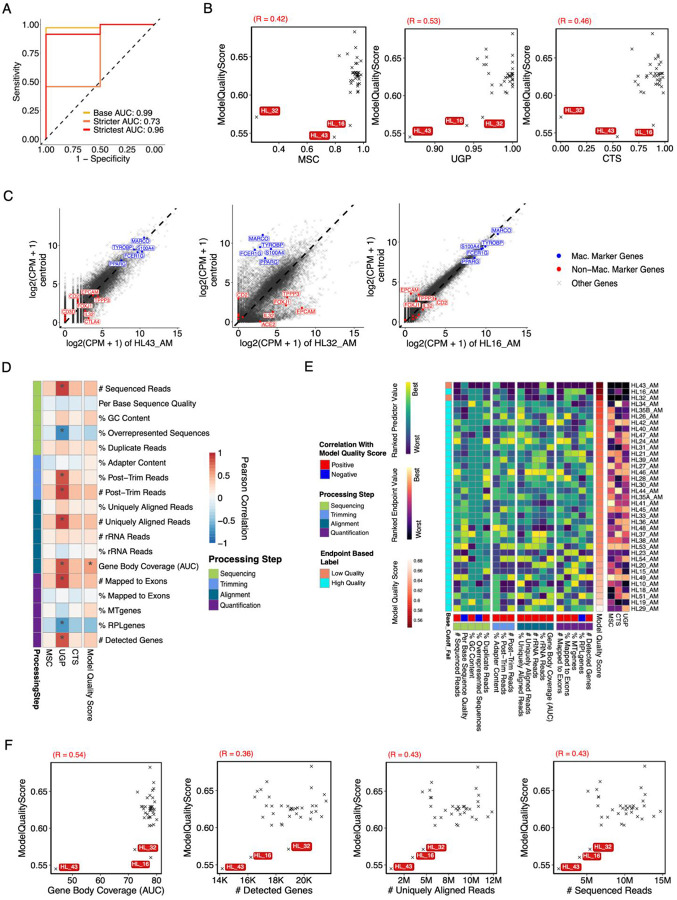
Validation of Random Forest Model on an Independent Test Set. (**A)** Receiver Operating Curve (ROC) showing the performance of the base, stricter, and strictest random forest models trained on the SCRIPT dataset when tested on the Lung Transplant dataset. **(B)** Scatterplots showing the relationships between each endpoint metric versus base model quality scores when tested on the Lung Transplant dataset. **(C)** Scatterplots showing gene expression values from the three samples with the lowest base model quality score in the Lung Transplant dataset compared against a centroid generated by averaging all other samples. Macrophage marker and non-macrophage marker genes are labeled and colored red and blue, respectively. **(D)** Heatmap showing the Pearson correlations of pipeline QC versus endpoint metric and base model quality score for the Lung Transplant Dataset. **(E)** Heatmap showing ranking of pipeline QC and endpoint metrics across samples in the Lung Transplant dataset ordered by base model quality score. Rank order of pipeline QC was determined by the correlation with model quality score in **D**. The label based on the endpoint cutoff (see Supp. Figure 5) is also given. **(F)** Scatterplots showing the relationship between the base model quality score and select pipeline QC metrics.

To assess the generalizability of our model, we applied it to an independent test set consisting of 37 alveolar macrophage samples collected from lung transplant donors and recipients (44) As is common with data obtained from a third party, we did not have access to Experimental QC for the Lung Transplant dataset and the FASTQ files were pre-filtered; however, we re-ran our entire pipeline anyway, including trimming, which led to unusually high *% Post-Trim Reads*. Moreover, although both datasets reported RNA-seq data on alveolar macrophages, they differed considerably in the distribution of QC metrics: the Lung Transplant dataset exhibited lower *# Sequenced Reads* but higher *% Uniquely Aligned Reads*, *% Mapped to Exons*, and *# Detected Genes*, though the *# Uniquely Aligned Reads and # Mapped to Exons* was more comparable to SCRIPT and highly correlated (Fig. S5A–B, Supplementary Table 9). Nevertheless, we applied a similar endpoint metric cutoff and identified only 2 samples (HL_32 and HL_43) that qualified as low quality and were outliers on the PCA of gene expression (Fig. S5C–F). When we tested our random forest models, we found that the models trained on the base and strictest cutoffs for the SCRIPT dataset performed best on the Lung Transplant dataset (Fig. 5A, Supplementary Table 10). The quality score generated by the base model was significantly correlated with all endpoint metrics; HL_32 and HL_43 along with HL_16 scored the lowest among samples (Fig. 5B). While HL_43 and HL_32 showed a distinct discordance in gene expression when plotted against the dataset centroid, the issue with HL_16 was less obvious (Fig. 5C). However, these 3 samples had the lowest *# Sequenced Reads*, *# Uniquely Aligned Reads*, and *# Mapped Exons* suggesting that HL_16 was indeed a low-quality sample (Fig. 5D–E). As in the SCRIPT dataset, no one QC metric alone could predict sample quality; *AUC-GBC* was most highly correlated but would not have flagged HL_16 (Fig. 5F). In addition, *# Detected Genes, # Uniquely Aligned Reads*, and *# Sequenced Reads* were correlated to quality in both data sets. Based on our insights from model performance on the test set, we ran QC-DR with custom thresholds to identify the low-quality samples (Supplementary Table 11, Extended Data 2). Taken together, our results underscore the importance of a consistent, multi-variable and data driven approach to defining low-quality samples in clinical RNA-seq datasets.

## Discussion:

**Figure 6:**
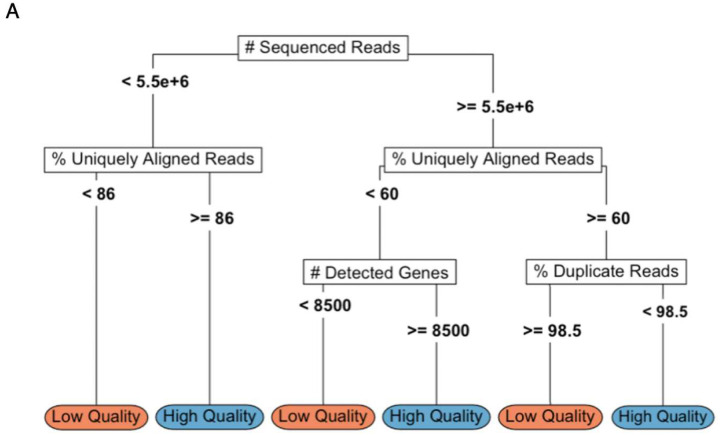
Proposed Simplified Decision Tree Model for Identifying Low-Quality Samples. **(A)** The performance of a simplified decision tree summarizing insights from the larger random forest model on the SCRIPT dataset. The label in the terminal node indicates whether the model predicts the sample to be low or high quality. The numbers in the bottom left and bottom right of each node show the number of low-quality and high-quality samples that fall into that node. Leaf node color indicates whether the model assigned samples within that node as low-quality (orange) or high-quality (blue). **(B)** The performance of the simplified decision tree described in **(A)** on the Lung Transplant dataset.

The large number of potential RNA-seq QC metrics, coupled with limited data on their relative utility, presents a significant challenge when conducting RNA-seq QC. To address this issue, we developed QC-DR, a tool that visualizes multiple QC metrics simultaneously and compare them to the background dataset. In the comprehensive analysis that followed, we assessed the relationship between 18 distinct QC metrics and their utility in predicting sample quality. Across 3 different stringency-based training sets and an independent test set, we found that *# Uniquely Aligned Reads*, *% Duplicate Reads, # Detected Genes, and AUC-GBC* were consistently highly weighted. However, no single metric alone could reliably identify low-quality samples, underscoring the need to consider multiple metrics in combination. Most QC metrics were correlated with better sample quality up to an inflection point, beyond which they had no impact. Since these relationships support binary thresholds, we present a simplified decision tree that accurately identifies low-quality samples in our training and test datasets (Fig. 6; Accuracy SCRIPT = 98%, Lung Transplant = 100%). Custom thresholds in QC-DR can be used to assist in implement this model. This decision tree represents an example of how to implement our insights into actionable QC filtering without significantly compromising predictive power.

Our finding that experimental QC do not correlate with final sample quality may be cause to re-think sample pipelines. Prior studies have examined the relationship between sample RNA concentration and statistics such as gene mapping and transcript alignment (45). Romero et. al. and Opitz et. al. characterized how RIN was associated with the degradation of different kinds of RNA transcripts (8,46). Jaffe et al. described the association of RIN with degradation of gene expression, and proposed a statistical framework, qSVA, to correct for RNA degradation bias (47). If a calculable RIN score or minimal RNA concentration had been used to discard samples before sequencing in the SCRIPT dataset, many usable samples would have been lost which translates to missing data points. Samples with low RNA fragment size did exhibit undesirable features such as high adaptor content, low post-trim reads, and high *% rRNA Reads*; however, this did not translate into a significant correlation with sample quality. It is important to note that our analysis only included samples that were sequenced as part of the SCRIPT study. We cannot know whether samples that were discarded based on experimental QC would have been low quality. Hypothetically, had these samples been included, we may have observed a higher correlation with sample quality. Another reason experimental QC metrics may not translate into sample quality is that non-optimal pooling of samples within a sequencing library may leads to under sequencing that compromises an otherwise unproblematic sample. Finally, there are issues that experimental QC cannot detect, such as cell contamination or low library complexity.

In this study, we introduce # Detected Genes and AUC-GBC as among the most useful individual QC metrics. This is significant as the utility of # Detected Genes for bulk RNA-seq QC has not previously been rigorously explored. Crucially, it is also one of the simplest metrics to calculate, by counting the non-zero values from the gene expression table. These findings align with existing literature. Illumina has questioned the reliability of RIN, one of the most common metrics used to disqualify samples, for indicating quality. GBC is more challenging both in computational complexity and time to calculate. Running the RSeQC approach with a wrapper script to combine results across samples required a few hours even using parallelization, though other algorithms may improve on this. The visualization provides an easy way to identify systemic RNA degradation in a given sample. Our contribution was to provide a method for quantifying the results across samples by calculating the area under the curve. In addition, QC-DR flags samples based on statistical Kolmogorov Smirnov (KS) test for determining if a sample’s curve significantly varies from the batch’s median. The success of # Detected Genes and AUC-GBC is likely due to their correlation with several other metrics and the fact that they incorporate both the concepts of transcriptome coverage and library complexity. While these variables do not stand alone in predicting sample quality, our results suggest they are consistently informative of sample quality between datasets.

Other approaches have been proposed for assessing sample quality in RNA-seq datasets. Wang et. al proposed an alternative to RIN that estimates RNA integrity using alignment information (39). GC content and different trimming criteria have been shown to affect gene detection and read mapping, but without providing guidance on how these metrics should be used for QC (48,49). Albrecht et al. developed a random forest model, SeqQScorer, that assigns RNA-seq samples quality scores based on features generated from FASTQ files, mapping information, and genomic location information (50–52). However, the intuition behind SeqQScorer and whether it applies to other studies is unclear. When we applied QC-DR to a large clinical RNA-seq dataset, we did not observe a uniform presentation of low-quality samples. Instead, we found samples had many distinct patterns of QC failure which we attribute to divergent sources. Reinforcing this idea, although many QC metrics correlated significantly with three distinct aspects of RNA-seq quality, numerous high-quality samples still exhibited poor values for these same QC metrics. By training a random forest model that integrated information from multiple QC metrics simultaneously, we were able to integrate a diverse set of metrics and their interactions to classify samples accurately. When this model was applied to an independent test set of alveolar macrophages from lung transplant patients, it was able to overcome the considerable differences in QC metric ranges and distributions between the two datasets to achieve accurate classification. Collectively, these results highlight the importance of considering multiple metrics when conducting QC and underscored the value of QC-DR’s multifaceted QC approach.

A primary limitation of this study was the scope of the data analyzed. Both the training and testing RNA-seq datasets were derived from alveolar macrophages using paired-end RNA-seq protocols on Illumina sequencers. For this reason, there may be some concern about whether our results are generally applicable. However, the SCRIPT and Lung Transplant datasets exhibited great variability in key QC metrics used by the random forest model. Despite this variability, the base model performed well on the test set and our analysis identified patterns supported by findings from previous research, including studies on FFPE samples and the ENCODE database (39,51). Thus, we have increased confidence that our specific insights will hold true. In any case, QC-DR provides a novel and unique tool that can be applied to any RNA-seq dataset. Moreover, the overlap in cell type enabled us to use the same endpoint metrics on both datasets for a unified concept of quality. While what constitutes acceptable RNA-seq quality may depend on the specific goals of the experiment, we chose our endpoint metrics to capture distinct sample characteristics that are intuitively important for quality (MSC = similarity; CTS = contamination; UGP = complexity). While *% Uniquely Aligned Reads and # Uniquely Aligned Reads* were of approximately equal importance in the SCRIPT dataset, the importance of absolute number was heightened in the Lung Transplant dataset where percent was high for all samples and sequenced reads were low across the dataset. Our random forest model was flexible enough to account for this distinction, and we demonstrate in our decision tree how using percent in combination with *# Sequenced Reads* can cover both cases. Other RNA-seq studies may raise new concepts of quality based on the specific experimental design and biological question. Moreover, changes in technology may change the specific thresholds, but the relevant metrics will likely remain constant. Our study serves as a blueprint for others to establish and justify their own quality control criteria

## Conclusion:

In summary, our work emphasizes the complexity inherent in RNA-seq QC and the need for approaches that integrate a broad array of QC metrics. QC-DR addresses this challenge by enabling simultaneous consideration of multiple QC metrics from across the entire sequencing process, including novel metrics for detection of genes and gene body coverage. Our findings show that the insights that can be gleaned from individual metrics is limited, and that combining multiple indicators, through qualitative or machine learning approaches, yields more accurate sample quality classification. Our study focuses on key QC metrics rather than assessing an exhaustive list; further investigation may be warranted on QC metrics we overlooked or novel metrics yet to be developed. Future studies building automated pipelines that incorporate QC-DR and machine learning approaches may facilitate real-time quality assessment, streamlining decisions on sample retention or exclusion. This would have particular value in clinical and diagnostic settings, where rapid and accurate identification of low-quality samples is essential.

## Methods:

### SCRIPT RNA-seq data:

Raw FASTQ files for 252 RNA-seq samples of alveolar macrophages isolated from mechanically intubated pneumonia patients were analyzed). Samples were sequenced across 11 batches. Samples were named based on the batch they belonged to and their sample number within that batch (e.g., sample B11_02 is the second sample from batch 11). These samples were collected from the *Successful Clinical Response in Pneumonia Therapy (SCRIPT)* project (IRB: STU00204868, dbGaP: phs002300.v1.p1). Details of sample collection and processing are described in Grant et al. 2021 (41). *# Total Cells*, *# Live Cells*, and *% Viability* were measured using a Nexcelom Cellometer. The *# Sorted Macrophages* was determined during cell sorting using a K2 FACS Aria III SORP instrument. *RIN*, *RNA Concentration*, and *RNA Fragment Size* were measured using the Agilent TapeStation 4200 with High Sensitivity RNA tapes. Samples with undetectable RIN were given N/A values. Libraries were prepared using the QIAGEN RNeasy Plus Micro Kit. After measuring *Sample Volume*, sequencing was performed using a full-length protocol with rRNA depletion.

### Lung Transplant Dataset:

Previously trimmed FASTQ files, comprising a dataset of 37 bulk RNA-seq samples (including a pair of technical replicates) of alveolar macrophages isolated from whole lung tissue of lung transplant recipients with pulmonary fibrosis and healthy donors, were analyzed. Details of sample collection and processing were described in Reyfman et al. 2019 (44) and obtained from the *Single Cell Analysis of Pulmonary Fibrosis* project (dbGaP: phs001750.v1.p1).

### Computational Pipeline for Processing RNA-seq and Generation of QC Metrics:

FastQC was used to calculate *# Sequenced Reads*, *% Overrepresented Sequences (Pre-trim)*, and *% Adapter Sequences (Pre-trim)* from these initial FASTQs for use in QC-DR. Next, adapters and low-quality reads were removed using BBDuk (53), and FASTQC was reapplied to the resulting trimmed FASTQs to calculate the *# Post-Trim Reads*, *% Post-Trim Reads*, *% Adapter Content (Post-Trim), % Overrepresented Sequences (Post-Trim), Per Base Sequence Quality, and % GC Content*. Trimmed reads were aligned to the human genome assembly hg19 using STAR (30) to generate data on *# Uniquely Aligned Reads, and % Uniquely Aligned Reads*. *# rRNA Reads* and *% rRNA Reads* were calculated by quantifying the number of overlapping reads between each BAM file and reference rRNA coordinates extracted from a BED file of rRNA loci using samtools (54). As a measure of complexity, *% Duplicate Reads* was calculated by Picard using the BAM files (55). Gene body coverage distributions were created by applying the geneBody_coverage.py function from RseQC to these BAM files and normalizing (0–1) within each sample (33). *Gene Body Coverage (AUC)* was calculated by summing all bin values and dividing by the number of bins (100). BAM files were then mapped to the genome using HTSEQ2 (31) and data on the *# Mapped to Exons*, *% Mapped to Exons*, *%MTgenes*, and *%RPLgenes*, were collected. The histogram of gene expression was generated by determining the number of genes with expression value in each bin range and the count of genes with value > 0 was calculated for the *# Detected Genes*. Complete information on what QC metrics were collected from each step is summarized in Table 2. For analysis, *Post-trim* values were used for *% Overrepresented Sequences* and *% Adapter Sequences* in the SCRIPT dataset, while *Pre-trim* values were used for the Lung Transplant dataset which was trimmed previously. Relatedly, *Overrepresented Sequences (Post-trim)* and *% Adapter Sequences (Post-trim)* were assigned zero values in the QC-DR input to represent these missing values in the Lung Transplant dataset.

### Quality Control Diagnostic Renderer (QC-DR):

QC-DR was developed in Python and was made publicly available on GitHub (40). To use QC-DR, users supply a query table of QC metrics as input to the main function QCDR_main, using either its Unix or Python interface. This query table contains the following columns*, Sample, Batch, # Sequenced Reads, # Post-Trim Reads, % Overrepresented Sequences (Pre-trim), % Adapter Content (Pre-trim), % Overrepresented Sequences (Post-trim), % Adapter Content (Post-trim), # Uniquely Aligned Reads, # rRNA Reads, # Mapped to Exons*. Of these, *Sample* must be filled while the other fields may be left blank, in which case the plots which require those data will be skipped. QC-DR uses percentile-based thresholding to identify and flag samples with aberrant values. To account for the non-normal distributions of many QC metrics, bootstrap sampling with replacement was used to generate a normal distribution of sample means. Samples which fall outside the specified percentiles of this estimated distribution were flagged as outliers. Optional tables may be supplied to QC-DR for additional functionality. If a reference table is supplied, QC-DR will use these data as the background for visualization and statistical inference. When the reference table is not supplied, the query table is used for these functions instead. Given a histogram of gene expression, QC-DR will generate an additional visualization of the gene expression distribution (an auxiliary function is available to generate a histogram from the raw count table if needed) and will flag samples based on their *# Detected* Genes. If a gene body coverage table is supplied, QC-DR will plot the average gene body coverage for each sample and flag samples by comparing the gene body coverage sample of each sample to a computed average distribution using the KS-test statistic. The gene body coverage table was generated by applying the geneBody_coverage.py function from the RseQC software to BAM files, 0–1 normalizing their outputs, and combining them into a single table. To further assist the user, example input and output files used to generate the data in this manuscript were packaged within the software on GitHub and included in the additional files (See Additional Files 2–6).

### Clustering of QC-DR Results:

SCRIPT samples were categorized into two groups, samples which failed only one QC metric and samples that failed multiple metrics. Each of these two groups was hierarchically clustered based on the flag vectors for each sample (0 = pass; 0.5 = warn; 1 = fail) with complete linkage and Euclidean distance. Three representative samples exhibiting distinct QC failure patterns, along with three samples that passed all QC metrics, were selected for comparative visualization across the metrics used by QC-DR. For further analysis, read counts from all SCRIPT samples were CPM-normalized and log-transformed before embedding them into a principal component analysis (PCA) for visualization. A computed centroid sample was produced by averaging the expression levels by gene across all samples and used to compare with global gene expression for the example samples. The macrophage marker genes and marker genes for other cell types used within these figures were selected based on literature review (Supplementary Table 7). All plots were created using ggplot2 in R.

### Calculation of Endpoint Metrics:

The Mean Sample Correlation (MSC) quantifies the similarity of a sample’s gene expression profile to a computed centroid sample. Let *x* represent a vector of gene expression values for an individual sample, and $\bar{x}$ denote the centroid vector, calculated as the mean gene expression for each gene across all samples. The MSC for each sample was then computed as the Pearson correlation between the sample vector *x* and the centroid vector $\bar{x}$. Thus, an MSC of 0 represents a sample with no correlation to the centroid and 1 represents a sample that is perfectly correlated to the centroid. This metric was designed to reflect the quality of RNA-seq samples with the assumption that samples suffering technical variance would exhibit lower correlation to $\bar{x}$ (Equation 1):

(1) $$MSC=\text{corr}(x,\bar{x})$$

The second endpoint metric, Unique Genomic Positions (UGP), measures genome coverage to assess whether an RNA-seq sample demonstrates sufficient complexity. Let *U* be the total number of unique genomic positions to which reads align, determined with the makeTagDirectory command in HOMER (56). An upper limit, *Lim*, representing the expected number of unique positions for a well-sequenced sample. For UGP estimation for the SCRIPT dataset, this was defined as *Lim* = 2 × 10^6^. For UGP estimation for the Lung Transplant dataset, this was defined as *Lim* = 5 × 10^6^. UGP was calculated as the logarithmic ratio of *U* to *Lim* with *U* capped at *Lim* to ensure the metric remained bounded between zero and one, where 0 represents sample with no aligned reads and 1 represents a well-sequenced sample. The calculation is expressed as (Equation 2):

(2) $$UGP=\frac{log_{10}(min(U,Lim))}{log_{10}(Lim)}$$

The third endpoint metric, Cell Type Score (CTS), was designed to measure the levels of unexpected transcripts, such as those expressed in non-macrophage cells (e.g., T cell gene CD3), relative to known alveolar macrophage genes (e.g., MARCO). This metric reflects the intuition that high quality samples should exhibit low expression of contaminating genes compared to marker genes. The selection of *m* alveolar macrophage marker genes and *c* contaminating genes was guided by a literature review, summarized in Supplementary Table 7. Let *M*_*i*_ denote the expression level of the *i*-th macrophage marker gene and *C*_*j*_ denote the expression level of the *j*-th contaminating gene, and. The CTS statistic was calculated as follows (Equation 3):

(3) $$CTS=\frac{\frac{1}{m}\sum_{i=1}^{m}M_{i}}{\frac{1}{m}\sum_{i=1}^{m}M_{i}+\sum_{j=1}^{c}C_{j}}$$

This formulation ensures that the CTS reflects the balance between contaminating and marker gene expression within each sample, bounded between zero and one, where 0 represents a sample which expresses no marker genes, and 1 represents a sample which expresses no contaminating genes. The correlation between CTS and other endpoint metrics was assessed using the Pearson method. Additionally, scatterplots of endpoint metrics were generated using ggplot2 in R (57).

### Relationships between QC Metrics:

To assess the relationships between experimental QC metrics, pipeline QC metrics, and endpoint metrics, Pearson correlation coefficients and their corresponding p-values were calculated between pairs of variables. These variable-variable associations were visualized by heatmaps created through the pheatmap package. The significance of each association was corrected for multiple comparisons within plots using the Benjamini-Hochberg (BH) adjustment. Selected variable-variable relationships were visualized using scatterplots.

### Training and Assessment of Random Forest Classifier:

SCRIPT samples were classified into high- and low-quality samples for model training based on the endpoint metrics. Three thresholds were defined 0.88, 0.9, and 0.91 corresponding to the base, stricter, and strictest cutoffs. Samples that fell below the cutoff in at least two out of three metrics were labeled “low-quality” and visualized with Euler plots using the *eulerr* R package (58). An independent random forest model was trained for each stringency cutoff based on the 18 pipeline QC metrics as predictors using the *randomForest* package in R (59). To estimate the models’ performance on unseen data, a quality score was calculated for each sample based on the proportion of out-of-bag (OOB) trees, those that did not observe that sample during training, that classified the sample as “high quality”. Then, AUC for each model was measured using the OOB quality score with the ROCR and pROC R packages (60). As a comparison, a logistic regression classifier was trained for each of the individual QC metrics and performance was evaluated by the AUC of the held-out samples from 5-fold cross-validation.

### Assessment of Variable Importance for the Random Forest Model:

Three methods were used to assess variable importance in the three random forest models. The Gini Purity method quantifies the reduction in Gini impurity at each node when a variable was randomized, estimating its contribution to model accuracy (61). The Variable Selection method measures the proportion of trees that include the variable. Finally, the Accumulated Local Effects (ALEs) estimates the impact of individual variables on the quality score based on a recently proposed approach (62). First, the model is decomposed into composite functions for each variable *x*_*i*_ using the methodology of ALEPlot (63). Then, each function *f*_*i*_(*x*_*i*_) is averaged across the values of *x*_*i*_ for all *n* samples in the dataset (Equation 4).

(4) $$\text{ALEAvgImp}(x_{i})=\frac{1}{n}\sum_{j=1}^{n}|f_{i}(x_{ij})|$$

The ALE approach was also used to visualize how changes to the value across the range of a given variable affects the model quality score and plotted with ggplot. Similarly, the ALE function was adapted to calculate how the interaction between pairs of variables affects the model quality score. The 2-dimensional ALE plots were visualize using base R.

### Validation of the Random Forest Classifier on an Independent Dataset:

Differences in the distributions of variables between the SCRIPT and Lung Transplant datasets were created in R. Endpoint metrics for samples within the lung transplant dataset were calculated using the same procedure as the SCRIPT dataset and samples were classified as “low quality” if they were below the base cutoff (0.88) in at least two metrics. The three random forest models trained on the SCRIPT dataset based on different stringencies were used to calculate a model quality score (0 = lowest quality; 1 = highest quality) and derive the AUC. Scatterplots of sample gene expression compared to centroid; relationships between QC metrics, endpoint metrics, and quality score; and PCA were plotted as described above for the SCRIPT dataset. A heatmap showing the relationships between QC metrics, endpoint metrics, and model quality scores was generated using pheatmap. Within this heatmap, the values of the QC and endpoint metrics were ranked as best to worst based on the association with quality in the SCRIPT dataset (i.e. the best rank was given to the highest value in a metric that was positively correlated with quality and the lowest value in a metric that was negatively associated with quality).

### Use of Large Language Models:

Use of the large language models Chat-GPT, Claude, and Le Chat were used to increase the efficiency of code.

## Supplementary Material

1 **Supplementary Figure 1. QC-DR Overview: (A)** Overview of the QC-DR workflow. **(B)** Summary heatmap of QC metrics across RNA samples generated by QC-DR for Batch 11 of the SCRIPT dataset. Each row represents an individual RNA sample, and each column represents a different QC metric. Gray cells indicate a pass for that metric, while yellow and red cells denote warn and fail flags, respectively. **(C)** QC-DR sample report of a poor-quality RNA-seq sample. Refer to Fig. 1A for a full description of QC-DR sample reports.
**Supplementary Figure 2. Examples of Low-quality Samples in SCRIPT Dataset. (A)** Heatmap showing quality flags assigned in SCRIPT samples assigned at least one by the QCDR (n = 102). **(B)** Distributions of experimental QC metrics in the SCRIPT dataset with high- and low-quality examples labelled from Figure 2. **(C)** Bar plots displaying experimental QC metrics for example high- (light blue) and low-(salmon) quality samples. Bar outlines indicate whether the metric was a pass (blue), warn (yellow), or fail (red).
**Supplemental Figure 3. Relationship of Experimental QC, Pipeline QC, and Endpoint Metrics. (A)** Heatmap of Pearson correlations between the experimental versus pipeline QC metrics in the SCRIPT dataset. * indicates statistically significant correlations after multiple hypothesis testing correction (p adj. < .05). **(B)** Scatterplots depicting key relationships between select experimental and pipeline QC metrics in the SCRIPT dataset. **(C)** Distributions of endpoint metrics in the SCRIPT dataset. with high- and low-quality examples labelled from Figure 2. **(D)** Scatterplot showing the relationships between CTS and MSC with an added color scale for UGP. **(E)** Heatmap displaying the Pearson correlations between MSC, UGP, and CTS **(F)** Box and whisker plot showing the distributions of endpoint metrics grouped based on their RIN values. Boxes show the 1^st^ quartile, median, and 3^rd^ quartile for each endpoint metric, while whiskers denote 1.5 × interquartile range (IQR). **(G)** Scatterplots depicting select experimental QC versus endpoint metrics. For all scatterplots, the correlation coefficient is given on the top left of each plot.
**Supplemental Figure 4. Classifying RNA-seq Quality with a Random Forest Model using Pipeline QC Metrics. (A)** Histograms displaying the distribution of the endpoint metrics for the SCRIPT dataset. Cutoffs for the base (0.88), stricter (0.9), and strictest (0.91) models are depicted with dashed vertical lines **(B)** Euler diagrams showing the number of samples in the SCRIPT dataset that are below the cutoffs for the three endpoint metrics at each stringency. **(C)** Heatmaps showing the variable importance scores for each pipeline QC metric for the stricter and strictest models based on Gini Purity, Variable Selection, and Accumulated Local Effects (ALE). Cells are colored based on the min-max normalized estimated effect, while the absolute values are given. **(D)** Heatmap showing the performance of the base, stricter, and strictest models on the SCRIPT dataset when classification is evaluated based on each stringency. **(E)** Heatmap showing the estimated effect of variable interactions on the model quality score in the stricter and strictest models **(F)** Two-dimensional ALE plots showing the estimated effect of the interactions between *Gene Body Coverage (AUC)* and *# Sequenced Reads* on the base model quality score. Circles represent the actual sample values. **(G)** PCAs of gene expression values for SCRIPT dataset where samples are colored based on which stringency they are labelled as low-quality (left) or the quality score assigned by the base model (right).
**Supplemental Figure 5: Validation of Random Forest Model on an Independent Test Set**, (**A)** Box and whisker plots comparing the distribution of pipeline QC metrics between SCRIPT and Lung Transplant datasets. Boxes denote the 1^st^ quartile, median, and 3^rd^ quartile values. Whiskers show the 1.5 inter-quartile range (IQR). **(B)** Heatmap showing the Pearson correlation between pipeline QC metrics within the Lung Transplant Dataset. * indicates statistically significant correlations (p adj. < .05). **(C)** Histograms displaying the distribution of the endpoint metrics for the Lung Transplant dataset. The cutoff (0.88) is depicted with a dashed vertical line. **(D)** Scatterplot showing the relationships between CTS and MSC in the Lung Transplant dataset with an added color scale for UGP. **(E)** Euler diagrams showing the number of samples in the Lung Transplant dataset that are below the endpoint cutoffs. **(F)** PCA of gene expression values for the Lung Transplant dataset.

ADDITIONAL FILES:

Extended Data 1: QC-DR Example (SCRIPT). QC-DR Applied to an example batch of the SCRIPT Dataset with Default Settings.

Supplementary Table 1. Example Input File of QC Metrics for QC-DR Using SCRIPT Dataset

Supplementary Table 2. Example Gene Expression Distribution Input File for QC-DR Using SCRIPT Dataset

Supplementary Table 3. Example Gene Base Coverage Input File for QC-DR Using SCRIPT Dataset

Supplementary Table 4. Example QC Cutoffs Input/Output File for QC-DR Using SCRIPT Dataset

Supplementary Table 5. Example QC Flags Output File for QC-DR Using SCRIPT Dataset

Supplementary Table 6. Experimental QC, Pipeline QC, and Endpoint Metrics for SCRIPT Dataset

Supplementary Table 7. List and Source of Macrophage Marker Genes and Non-Macrophage Contaminating Genes

Supplementary Table 8. Random Forest Model Quality Score and Predictions for SCRIPT Dataset

Supplementary Table 9. Pipeline QC and Endpoint Metrics for Lung Transplant Dataset

Supplementary Table 10. Random Forest Model Quality Score and Predictions for Lung Transplant Dataset

Supplementary Table 11. Example Custom QC Cutoffs for QC-DR Using Lung Transplant Dataset

Extended Data 2: QC-DR Example (Lung Transplant). Example of QC-DR Applied to Lung Transplant Dataset Using Custom Cutoffs.

Supplementary Files

This is a list of supplementary files associated with this preprint. Click to download.

- ExtendedData1.pdf
- SupplementaryTable1.csv
- SupplementaryTable2.xlsx
- SupplementaryTable3.csv
- SupplementaryTable4.xlsx
- SupplementaryTable5.csv
- SupplementaryTable6.csv
- SupplementaryTable7.xlsx
- SupplementaryTable8.csv
- SupplementaryTable9.csv
- SupplementaryTable10.csv
- SupplementaryTable11.xlsx
- ExtendedData2.pdf

## Figures and Tables

**Table 1.** Explanation of Experimental QC

Experimental QC	Description	Source
**Sample Volume**	The volume of Bronchoalveolar lavage (BAL) fluid used for RNA-seq processing.	Technician
**RNA Integrity Number (RIN)**	An estimate of RNA quality based on the proportion of intact 28S and 18S ribosomal RNA, calculated using electropherogram analysis.	Gel Electrophoresis
**RNA Concentration**	The quantified amount of total RNA present in the sample after purification, measured by fluorescence-based quantification or absorbance at 260 nm.	Gel Electrophoresis
**RNA Fragment Size**	The average length of RNA fragments in base pairs, measured by electropherogram analysis, which generates a size distribution curve of RNA molecules.	Gel Electrophoresis
**# Total Cells**	The total number of cells collected during sample acquisition, reported during fluorescence-activated cell sorting (FACS).	Fluorescence-activated Cell Sorting (FACS)
**# Sorted Macrophages**	The number of macrophages collected, based on gating strategies and fluorophore-labeled markers used in fluorescence-activated cell sorting (FACS).	Fluorescence-activated Cell Sorting (FACS)
**# Live Cells**	The number of live cells, identified by exclusion of viability dyes.	Fluorescence-activated Cell Sorting (FACS)
**% Viable Cells**	The proportion of *# Live Cells* to *# Total Cells*.	Fluorescence-activated Cell Sorting (FACS)

**Table 2.** Explanation of QC Metrics

QC Metric	Processing Step	Input	Description	Source
**# Sequenced Reads**	Sequencing	FastQ File	The total number of reads detected by the sequencer before trimming	FASTQC
**Per Base Sequence Quality**	Sequencing	Trimmed FASTQ file	The average Phred quality of each base read by the sequencer. Phred quality scores are determined by the confidence in the base pair call.	FASTQC
**% GC Content**	Sequencing	Trimmed FASTQ file	The percentage of sequenced bases which are guanine or cytosine	FASTQC
**% Duplicate Reads**	Sequencing	BAM file	Picard estimates % Duplicate reads by counting the number of reads which have the same starting base and ending base	Picard
**% Adapter Content**	Trimming	Trimmed FASTQ file	The percentage of known adapter sequences detected among sequenced reads. This metric is calculated pre (untrimmed) and post (trimmed) trimming. The latter value is used unless otherwise specified.	FASTQC
**% Overrepresented Sequences**	Sequencing	Trimmed FASTQ file	The percentage of reads determined by FASTQC to appear more frequently than expected by chance. This metric is calculated pre (untrimmed) and post (trimmed) trimming. The latter value is used unless otherwise specified.	FASTQC
**# Post-Trim Reads**	Trimming	Trimmed FASTQ file	The number of reads remaining after removing adapter sequences and poor quality reads	FASTQC & BBduk
**% Post-Trim Reads**	Trimming	Pre-trim FASTQ file & Trimmed FASTQ file	The percent of *# Post-Trim reads* over *# Sequenced Reads*	FASTQC & BBduk
**# Uniquely Aligned Reads**	Alignment	BAM file	The number of reads aligned only to one genomic location	samtools
**% Uniquely Aligned Reads**	Alignment	BAM file	The percent of *# Uniquely Aligned Reads over # Post-Trim Reads*	samtools
**# rRNA Reads**	Alignment	BAM file & BED file	The number of uniquely aligned reads overlapping annotated rRNA loci, determined using samtools with a BED file of rRNA regions as reference	samtools
**% rRNA Reads**	Alignment	BAM file & BED file	The percent of *# rRNA Reads* over *# Uniquely Aligned Reads*	samtools
**AUC-GBC**	Alignment	BAM file	The area under the curve (AUC) of the gene body coverage (GBC) plot. To calculate the statistical threshold for GBC in QC-DR, a Kolmogorov-Smirnov (KS) test is run comparing each sample to the median of the dataset.	RseQC + custom
**# Mapped to Exons**	Quantification	Count Table	The number of reads mapped to exonic regions of the genome.	HTSEQ2
**% Mapped to Exons**	Quantification	Count Table	The percent of # *Mapped to Exons* over *# Uniquely Aligned Reads*	HTSEQ2 / STAR
**%RPLgenes**	Quantification	Count Table	The number of read mapped to ribosomal protein genes over *# Mapped to Exons*	HTSEQ2
**% MTgenes**	Quantification	Count Table	The number of reads assigned to mitochondrial genes over *# Mapped to Exons*	HTSEQ2
**# Detected Genes**	Quantification	Raw Count Table	The number of unique genes with non-zero read counts	HTSEQ2

## Data Availability

QC-DR is available for download on GitHub (40). This software is platform independent and contains code written in Python and Unix. It is distributed using the MIT License. The processed data supporting the conclusions of this article are included within the article and its additional files. RNA-seq datasets used in analysis are available on dbGaP under the following projects: Successful Clinical Response in Pneumonia Therapy (SCRIPT), PI: Wunderink (phs002300.v1.p1) and Single Cell Analysis of Pulmonary Fibrosis, PI: Budinger & Misharin (phs001750.v1.p1).
