# Supplementary material for: Integration of Bulk RNA-seq Pipeline Metrics for Assessing Low-Quality Samples
Source: Res Sq. 2025 Jul 3:rs.3.rs-6976695. Preprint. [Version 1] doi: 10.21203/rs.3.rs-6976695/v1 (PMC12236924; doi:10.21203/rs.3.rs-6976695/v1)

Supplementary Figure 1

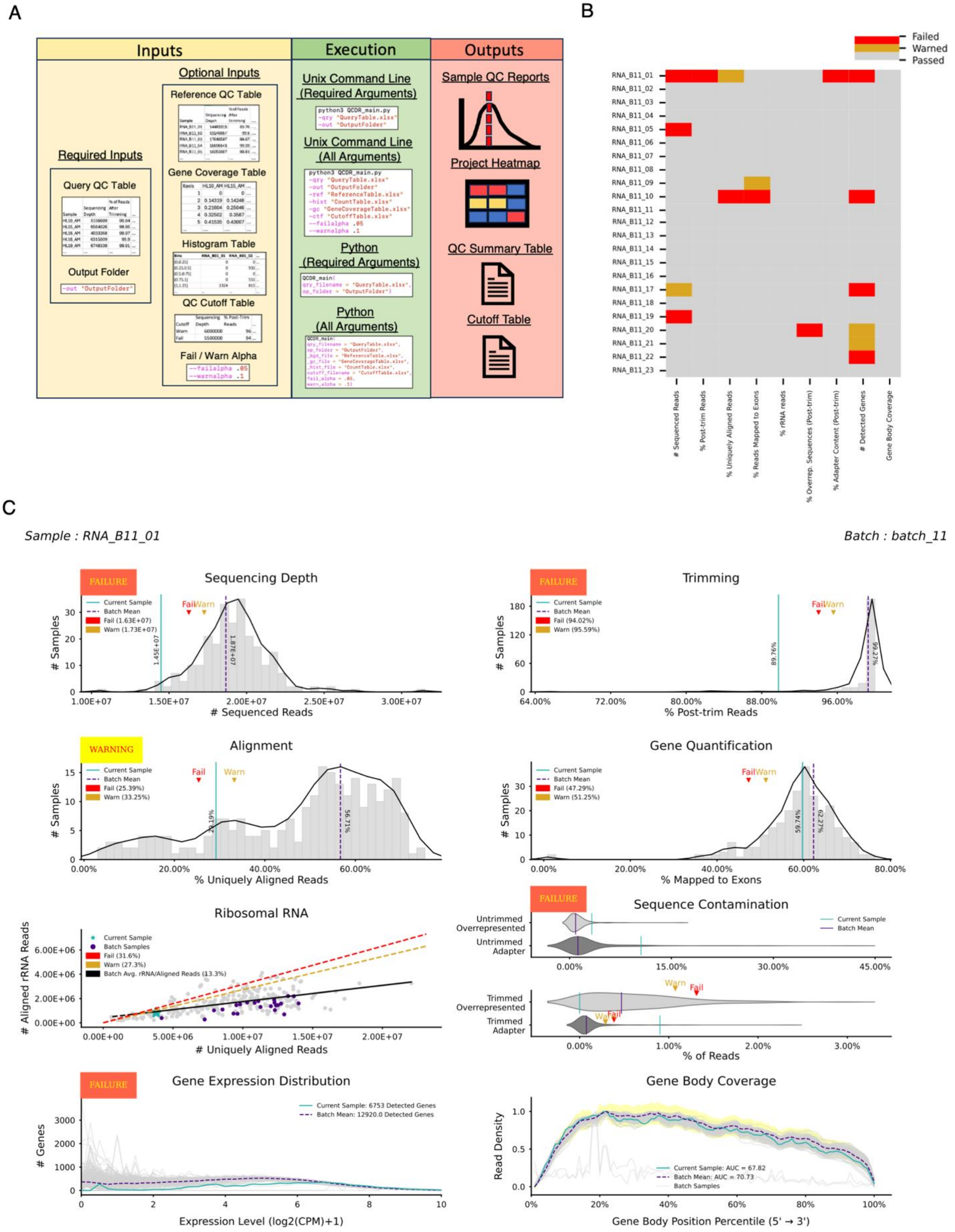

Supplementary Figure 2

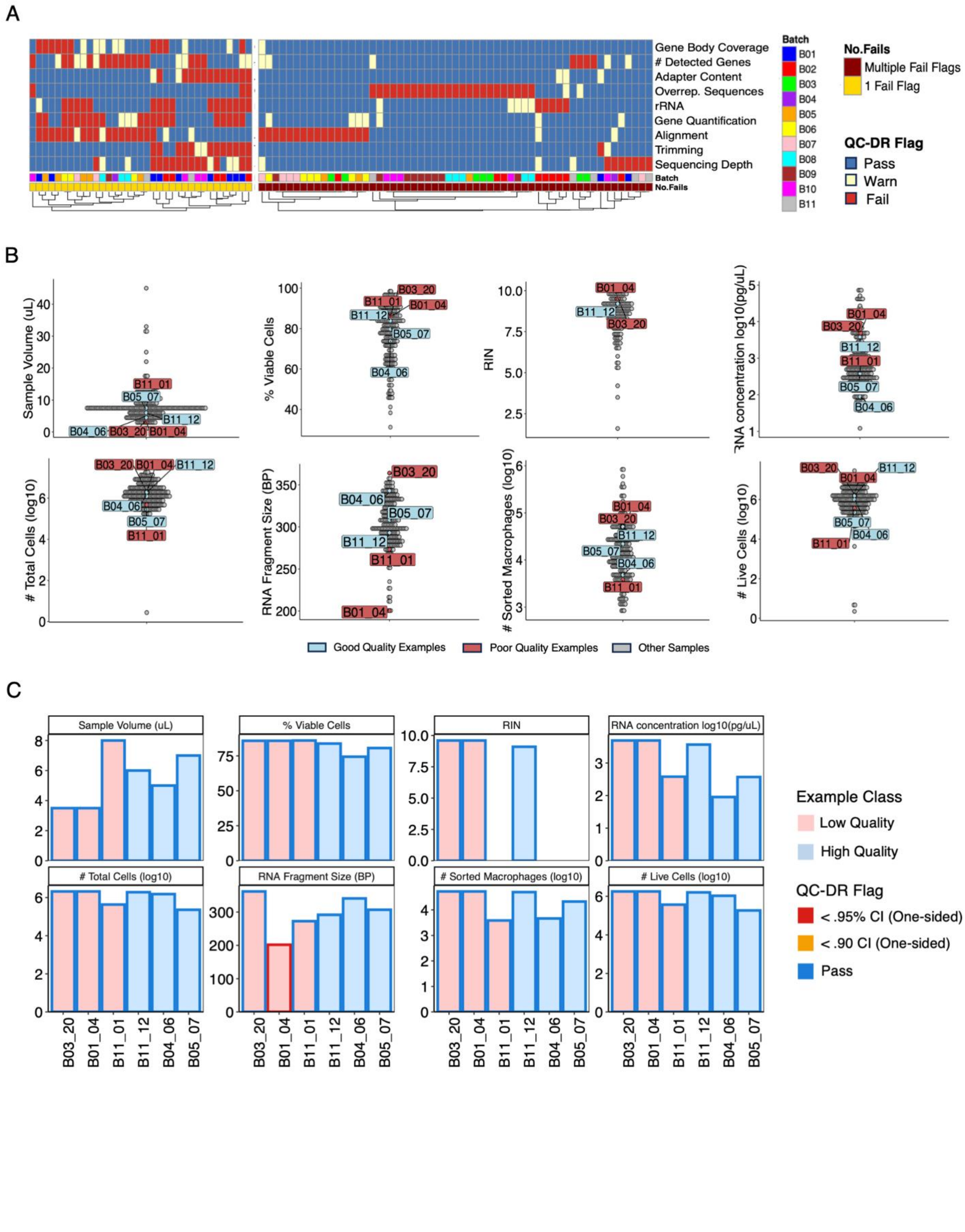

Supplementary Figure 3

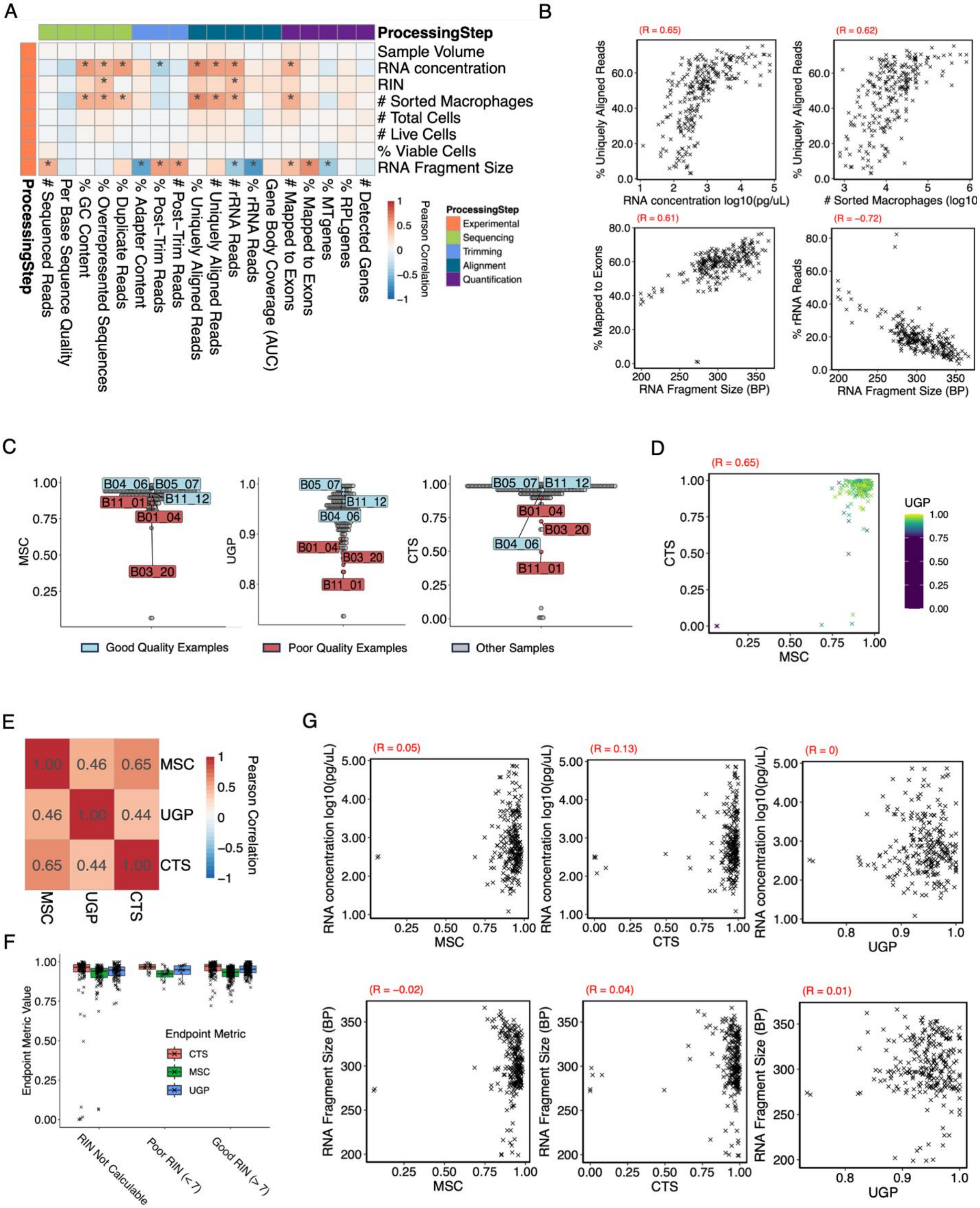

Supplementary Figure 4

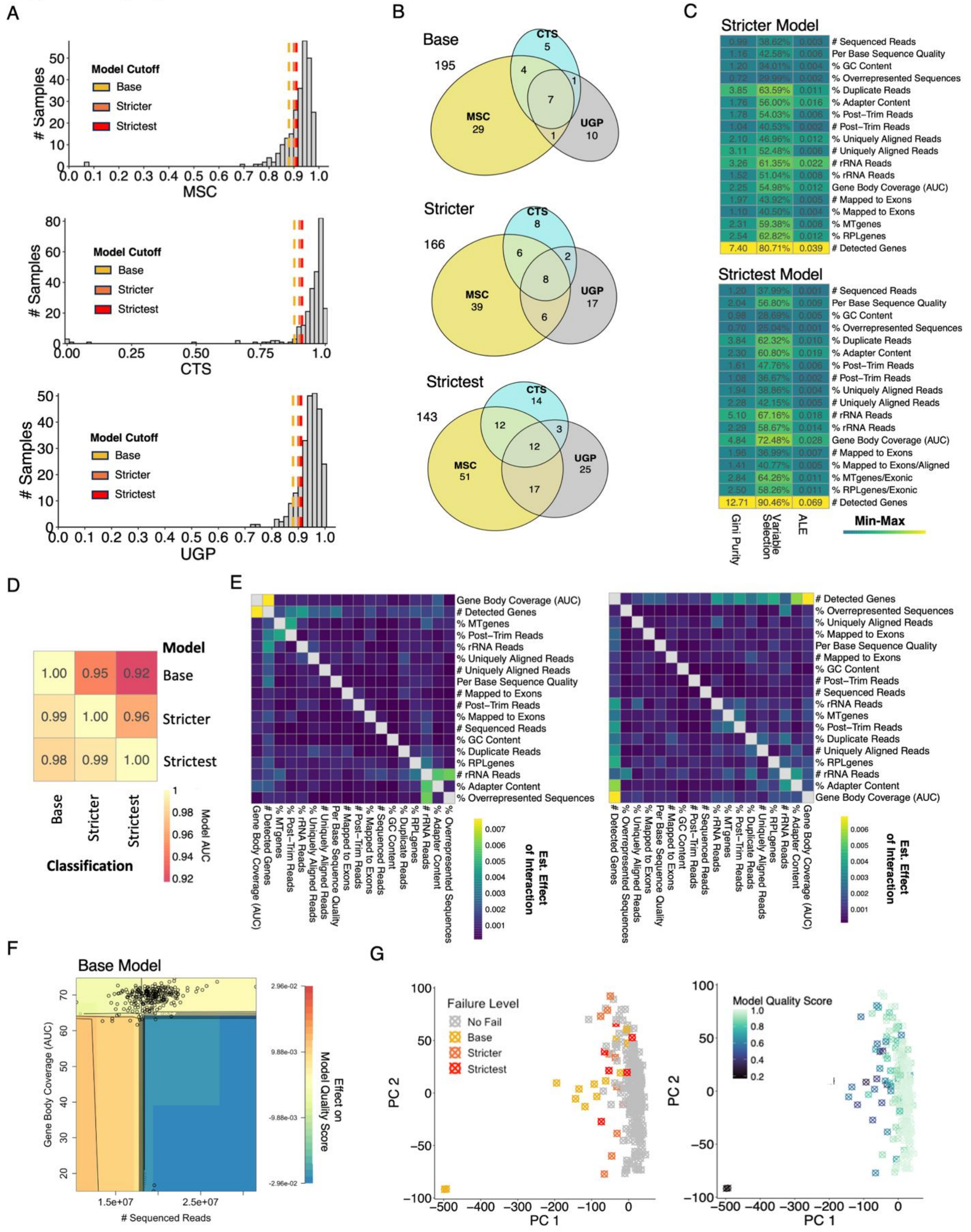

Supplementary Figure 5

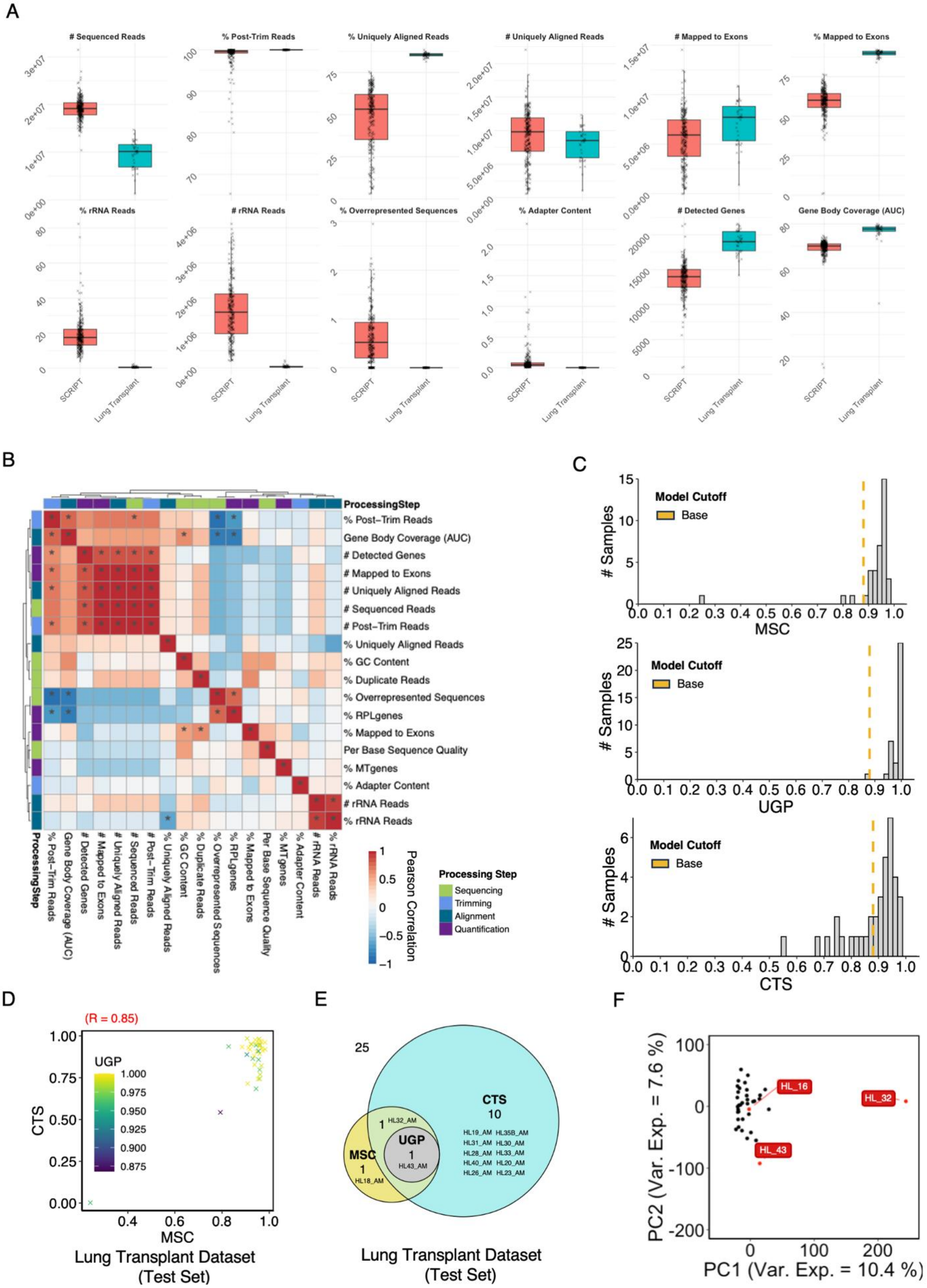

Supplement: 1 — Supplementary Figure 1. QC-DR Overview: (A) Overview of the QC-DR workflow. (B) Summary heatmap of QC metrics across RNA samples generated by QC-DR for Batch 11 of the SCRIPT dataset. Each row represents an individual RNA sample, and each column represents a different QC metric. Gray cells indicate a pass for that metric, while yellow and red cells denote warn and fail flags, respectively. (C) QC-DR sample report of a poor-quality RNA-seq sample. Refer to Fig. 1A for a full description of QC-DR sample reports. Supplementary Figure 2. Examples of Low-quality Samples in SCRIPT Dataset. (A) Heatmap showing quality flags assigned in SCRIPT samples assigned at least one by the QCDR (n = 102). (B) Distributions of experimental QC metrics in the SCRIPT dataset with high- and low-quality examples labelled from Figure 2. (C) Bar plots displaying experimental QC metrics for example high- (light blue) and low-(salmon) quality samples. Bar outlines indicate whether the metric was a pass (blue), warn (yellow), or fail (red). Supplemental Figure 3. Relationship of Experimental QC, Pipeline QC, and Endpoint Metrics. (A) Heatmap of Pearson correlations between the experimental versus pipeline QC metrics in the SCRIPT dataset. * indicates statistically significant correlations after multiple hypothesis testing correction (p adj. < .05). (B) Scatterplots depicting key relationships between select experimental and pipeline QC metrics in the SCRIPT dataset. (C) Distributions of endpoint metrics in the SCRIPT dataset. with high- and low-quality examples labelled from Figure 2. (D) Scatterplot showing the relationships between CTS and MSC with an added color scale for UGP. (E) Heatmap displaying the Pearson correlations between MSC, UGP, and CTS (F) Box and whisker plot showing the distributions of endpoint metrics grouped based on their RIN values. Boxes show the 1st quartile, median, and 3rd quartile for each endpoint metric, while whiskers denote 1.5 × interquartile range (IQR). (G) S [file NIHPPRS6976695V1-supplement-1.pdf]
